# Correction: Dynamic imaging of myelin pathology in physiologically preserved human brain tissue using third harmonic generation microscopy

**DOI:** 10.1371/journal.pone.0329374

**Published:** 2025-08-04

**Authors:** The PLOS One Editors

## Notice of Republication

This article [1] was republished on July 7, 2025, to address an issue identified post-publication.

Additionally, the spelling of the second individual in the 6th line of the Acknowledgments is corrected to Marko Popovic. Please download this article again to view the correct version.
