# Supplementary figures and images for: Dynamic imaging of myelin pathology in physiologically preserved human brain tissue using third harmonic generation microscopy
Source: PLoS One. 2025 Mar 31;20(3):e0310663. doi: 10.1371/journal.pone.0310663 (PMC11957345; doi:10.1371/journal.pone.0310663)

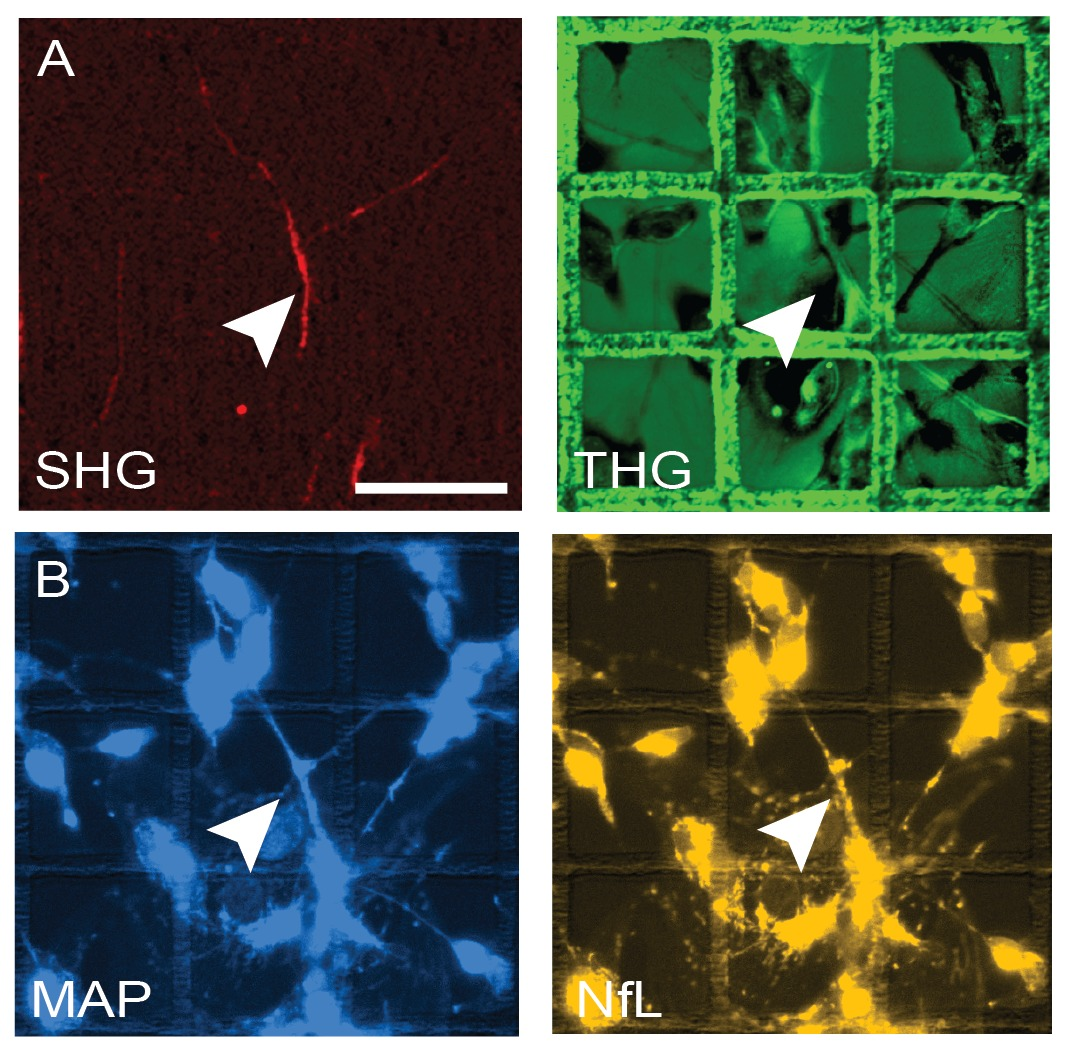

Supplement: Supplementary Fig 1 — (TIF) [file pone.0310663.s001.tif]
